# Long-term outcomes of intravitreal 0.19 mg fluocinolone acetonide implant in uveitic macular edema after prior local corticosteroid treatments

**DOI:** 10.1186/s12348-025-00558-7

**Published:** 2025-12-08

**Authors:** Ana Margarida Ferreira, Marta Inês Silva, Sónia Torres-Costa, Cláudia Oliveira-Ferreira, Ana Catarina Pedrosa, Luís Figueira, Joana Rodrigues Araújo

**Affiliations:** 1 Department of Ophthalmology, Local Health Unit of São João, Porto, Portugal; 2 Department of Surgery and Physiology, Faculty of Medicine, University of Porto, Porto, Portugal; 3 Unit of Pharmacology and Therapeutics, Department of Biomedicine, Faculty of Medicine, University of Porto, Porto, Portugal; 4 MedInUP - Center for Drug Discovery and Innovative Medicines, Faculty of Medicine, University of Porto, Porto, Portugal; 5 Department of Ophthalmology of São João Hospital, Avenida Prof. Hernâni Monteiro, Porto, 4202 – 451 Portugal

**Keywords:** Fluocinolone intravitreal implant, ILUVIEN, Macular edema, Intraocular inflammation, Non-infectious uveitis, Posterior segment

## Abstract

**Background:**

This study evaluates the long-term efficacy and safety of intravitreal 0.19 mg fluocinolone acetonide implant (FAi) in non-infectious UME, comparing it with prior sub-Tenon’s triamcinolone acetonide (STTA) and/or intravitreal dexamethasone implant (DEXAi).

**Methods:**

A retrospective review of patients with chronic non-infectious UME who received intravitreal FAi after STTA and/or DEXAi.

**Results:**

Thirteen eyes from 7 patients (69.2% female) were included, with a mean age of 65 ± 11 years. The median follow-up was 54 months before FAi (IQR: 43–65) and 47 months after FAi (IQR: 23–60). All eyes received at least one STTA, and 11 eyes received at least one DEXAi before FAi. The median number of injections before FAi was 4 (range: 2–8). A significant reduction in recurrences per year was observed after FAi (4.62 ± 1.9 before vs. 0.31 ± 0.5 after, Wilcoxon test, *p* = 0.001). Macular edema relapsed in 4 eyes (30.8%) between months 28 and 36. Central retinal thickness significantly decreased after FAi (474 ± 96 μm before vs. 305 ± 58 μm after, Wilcoxon test, *p* = 0.002). Ten eyes (76.9%) showed BCVA improvement (1–4 lines), and none lost visual acuity. All eyes were pseudophakic prior to FAi. At FAi, four of seven patients were on systemic anti-inflammatory therapy; 3 discontinued it, and none started new treatment after FAi. Five eyes were on topical IOP-lowering medication at the time of FAi; 2 required and increase in treatment, and 2 later underwent trabeculectomy. After FAi, only two eyes started IOP-lowering medication.

**Conclusions:**

In this case series with long-term follow-up, FAi for non-infectious UME was effective, safe, and reduced recurrences compared to other local corticosteroids.

## Background

Uveitic macular edema (UME) represents a prevalent complication of any type of uveitis. It is a leading cause of visual impairment in patients with intraocular inflammation, resulting from inflammatory breakdown of the blood-retinal barrier, increased vascular permeability, and subsequent intraretinal fluid accumulation. The management of UME remains a significant challenge, as the condition is frequently chronic and recurrent, requiring long-term strategies to control both inflammation and macular edema while minimizing treatment-associated complications [1].

Corticosteroids (CCT) remain the mainstay of treatment, suppressing the inflammatory response at multiple levels: inhibition of pro-inflammatory cytokines and mediators, strengthening of the blood-retinal barrier (by reducing endothelial cell activation and restoring tight junction integrity) and reducing immune-mediated retinal damage (with suppression of autoimmune response). Beyond their ability to reduce inflammation, corticosteroids directly mitigate macular edema by addressing the vascular and cellular mechanisms that contribute to fluid accumulation (reduction of vascular permeability, decrease in fluid accumulation and inhibition of retinal Müller cells which are involved in fluid balance and homeostasis) [2, 3].

CCT can be administered systemically (oral or intravenous), locally (periocular injections), or intravitreally, depending on disease severity and laterality. However, long-term systemic corticosteroid use is often limited by significant adverse effects, including hypertension, diabetes, osteoporosis, and adrenal suppression [4]. Similarly, repeated intravitreal or periocular steroid injections, while effective, carry risks such as ocular hypertension, cataract progression, and potential for rebound inflammation upon tapering [5, 6].

The intravitreal dexamethasone implant (DEXAi, 0.7 mg) has become an established short-acting corticosteroid option for the treatment of non-infectious uveitic macular edema, providing effective but transient control of inflammation and edema (typically duration of effect 3–4 months). Several studies have demonstrated its efficacy in improving both visual acuity and central retinal thickness; however, many patients experience recurrent edema requiring repeated injections, which increases treatment burden and the risk of ocular hypertension.^[1–4]^ [7–10].

To address these challenges, long-acting intravitreal steroid implants such as Iluvien (fluocinolone acetonide intravitreal implant, 0.19 mg) have emerged as an alternative, providing sustained corticosteroid delivery over an extended period (continuous microdosing for up to 36 months) and reducing the need for frequent retreatments. Iluvien, originally approved for diabetic macular edema (DME), has been increasingly recognized for its role in managing chronic and recurrent uveitic macular edema, offering prolonged disease control and potential visual benefits, representing a long-term alternative for patients with recurrent or chronic UME insufficiently controlled by shorter-acting corticosteroids [11, 12].

This study aims to evaluate long-term efficacy and safety of intravitreal 0.19 mg fluocinolone acetonide implant (FAi) (Iluvien^®^) in non-infectious UME and compare it with previous sub-Tenon’s capsule triamcinolone acetonide (STTA) injection and/or intravitreal dexamethasone implant (DEXAi).

## Methods

A retrospective, single center, observational study including adult patients with chronic macular edema secondary to non-infectious uveitis that were previously treated with STTA and/or DEXAi and later proposed to treatment with intravitreal FAi.

Chronic UME was defined as the accumulation of fluid within the retinal layers as cystoid spaces or diffuse retinal thickening or in the subretinal space between the neurosensory retina and retinal pigment epithelium, for more than six months (objectively assessed by central macular thickness (CMT) measurements) [13]. In the patients included in our cohort, chronic cystoid macular edema was defined as either persistent (unresponsive or poorly responsive) or recurrent (with persistent uveitis that relapsed in less than 3 months after the previous treatment) macular edema lasting ≥ 6 months despite previous local corticosteroid treatments (STTA and/or DEXAi).

Medical records since the uveitis diagnosis were reviewed for each patient. Data regarding demographic, clinical and imaging parameters were collected over the follow-up period, including age, gender, lens status, best-corrected visual acuity (BCVA, measured on a decimal scale and converted to LogMAR), intraocular pressure (measured with a Goldman applanation tonometer or iCare tonometer) and CMT and recurrence of macular edema assessed by spectral domain optical coherence tomography (SD-OCT) (Spectralis, Heidelberg Engineering, Heidelberg, Germany). The number of STTA and DEXAi administered before the FAi was determined, as well as the number of recurrences of macular edema per year. At the time of FAi implantation, intraocular inflammation was graded according to the Standardization of Uveitis Nomenclature (SUN) criteria, including anterior chamber cells (0–4+), flare (0–4+), and vitreous haze (0–4+). The uveitis was anatomically classified as anterior, intermediate, posterior, or panuveitis based on SUN definitions [5, 14].

Normally distributed data is reported as mean and standard deviation (SD) while non-normally distributed data is reported as median and interquartile range (IQR). Proportions are reported as absolute number and percentage. Kolmogorov-Smirnov and Shapiro-Wilk tests were used to assess whether each variable followed a normal distribution. The continuous variables were compared between the paired observations using the parametric paired t-test or Wilcoxon matched-pairs signed-rank test, for parametric and non-parametric variables, respectively. All statistical analyses were performed using SPSS version [IBM-SPSS^®^ for Windows^®^, version 30.0], and a two-tailed *p* < 0.05 was considered statistically significant.

## Results

Thirteen eyes from 7 patients (69.2% of females) were included. The mean age was 65 ± 11 years. The median follow-up was 54 months before FAi (interquartile range [IQR]: 43–65 months) and 47 months after FAi (IQR: 23–60 months). After FAi, 11 (84.6%) eyes were followed for more than 24 months and 9 (69.2%) eyes for more than 36 months. All eyes were pseudophakic prior to FAi. Table 1 presents demographic and clinical characteristics of our cohort. At the time of FAi injection, 9 eyes (69%) presented with inactive uveitis (0 cells, 0 flare), and 4 eyes (31%) had mild activity (≤ 1 + cells or flare). Vitreous haze was present (grade 1–2) in 2 cases (15%). No eyes exhibited severe inflammation at baseline. The indication for FAi was chronic and recurrent uveitic macular edema rather than acute inflammatory activity. Relapse was defined as the recurrence of any inflammatory activity in a previously quiescent eye, with deterioration in visual acuity or the need for rescue medications.

**Table 1:** Demographic and clinical characteristics

Parameter	*n* = 13
Age, years	65 ± 11
Gender, male/female	4 (30.8) / 9 (69.2)
Lens Status	
Pseudophakic	13 (100)
Uveitis Anatomic Group	
Panuveitis	4 (30.8)
Anterior Uveitis	2 (15.4)
Anterior and Intermediate Uveitis	2 (15.4)
Vasculitis	5 (38.5)
Systemic Diagnosis	
Idiopathic intraocular inflammation	11 (84.6)
Spondylarthritis	2 (15.4)
Follow-up; median [IQR]	
Before FAi	54 [43–65]
After FAi	47 [23–60]

FAi: Intravitreal 0.19 mg fluocinolone acetonide implant; IQR: Interquartile range

Table 2 demonstrates treatment profile of the patients in our cohort, including systemic therapy, previous corticosteroid treatment and fluocinolone acetonide effectiveness. Regarding systemic immunomodulatory (IM) therapy at FAi, 6 (46.2%) eyes were under treatment with 1 IM drug (one cyclosporine, one azathioprine and four methotrexate) and 4 (30.8%) eyes with 2 IM drugs (combination of adalimumab and methotrexate in 2 eyes and infliximab and methotrexate in 2 eyes). No eyes were under treatment with systemic corticosteroids. After FAi, 3 patients discontinued IM therapy (2 cases of idiopathic panuveitis and 1 patient with idiopathic vasculitis), and none started new treatment.

**Table 2:** Treatment characterization, including systemic therapy, previous corticosteroid treatment and Fluocinolone acetonide effectiveness

Parameter	*n* = 13	
Immunomodulatory Systemic Therapy		
None	3 (23.1)	
1 Drug	6 (46.2)	
2 Drugs	4 (30.8)	
Previous corticosteroid treatment		
Sub-Tenon Triamcinolone Injection	13 (100)	
Dexamethasone implant	11 (84.6)	
Number of injections (STTA + DEXAi) before FAi, median [range]	4 [2–8]	
Number of recurrences per year		
Before FAi	4.62 ± 1.9	*p* < 0.001^a^
After Fai	0.31 ± 0.5	
Macular Edema relapse after FAi	4 (30.8)	
IOP-lowering medication		
Prior do FAi	5 (38.4)	
Initiated after FAi	2 (15.4)	
Required additional medication	2 (15.4)	
Trabeculectomy	2 (15.4)	

^a^Wilcoxon Signed-Rank Test

STTA: Sub-Tenon’s capsule triamcinolone acetonide; DEXAi: Intravitreal dexamethasone implant; FAi: Intravitreal 0.19 mg fluocinolone acetonide implant; IOP: Intraocular pressure

All eyes (100%) received at least one STTA, and 11 (84.6%) eyes received at least one DEXAi before FAi, presenting a median duration of effect of approximately 2–3 months after STTA and 3–4 months after DEXAi. The median number of injections (STTA + DEXAi) before FAi was 4 (range: 2–8). The median interval between the last local corticosteroid (either STTA or DEXAi) and FAi injection was 6 months (IQR 4–8). At the time of FAi, 7 eyes (54%) had recurrent UME on OCT (repeated episodes separated by periods of inactivity without treatment for ≥ 3 months), 3 (23%) eyes presented a CRT reduction but fluid persistence (incomplete response), while 3 (23%) eyes presented resolved ME and were injected prophylactically approximately 4 months after the previous treatment due to a history of frequent relapses.

**Fig. 1:**
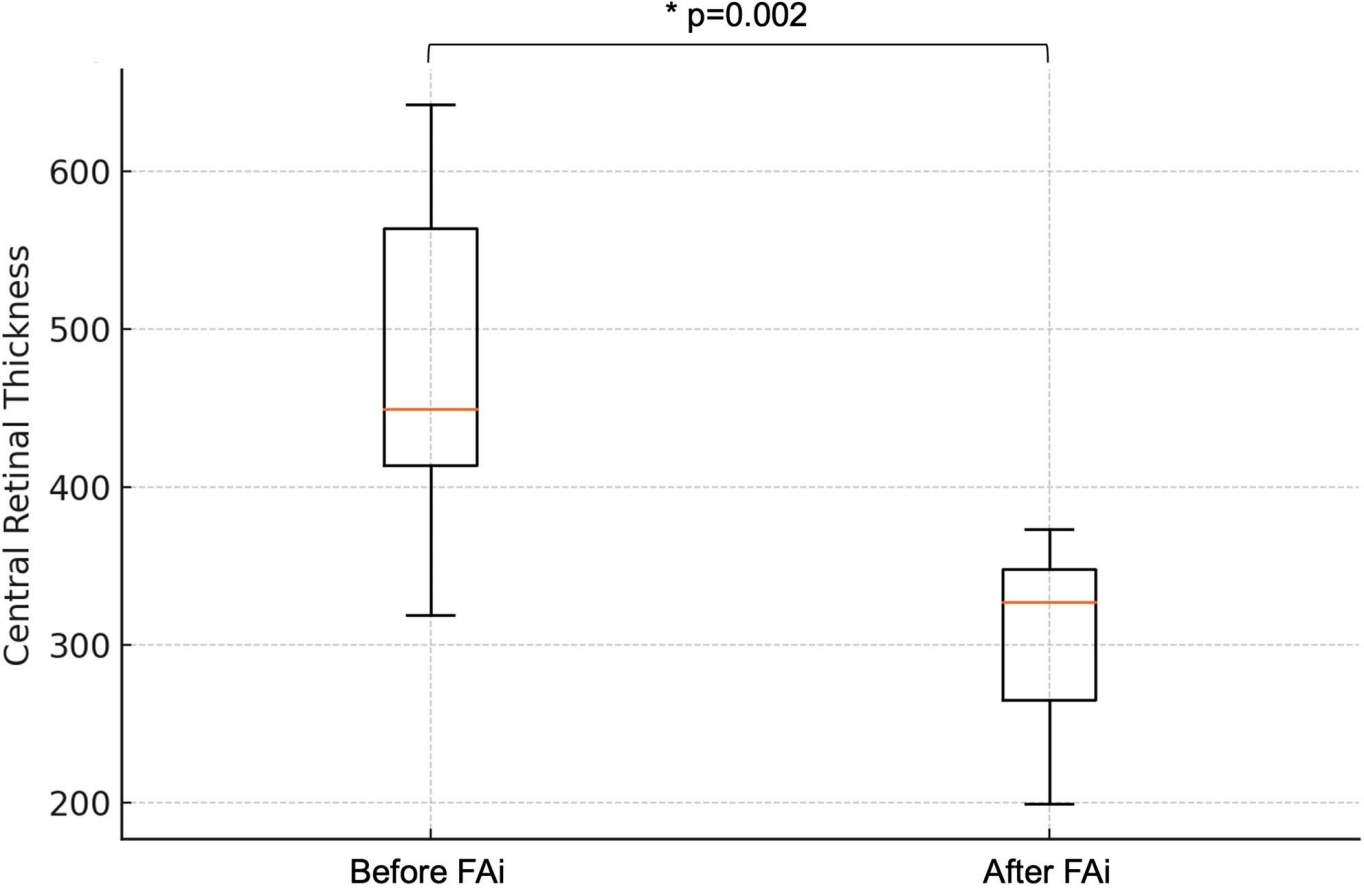
Boxplot of central retinal thickness before and after intravitreal 0.19 mg fluocinolone acetonide implant (FAi) (µm)

**Fig. 2:**
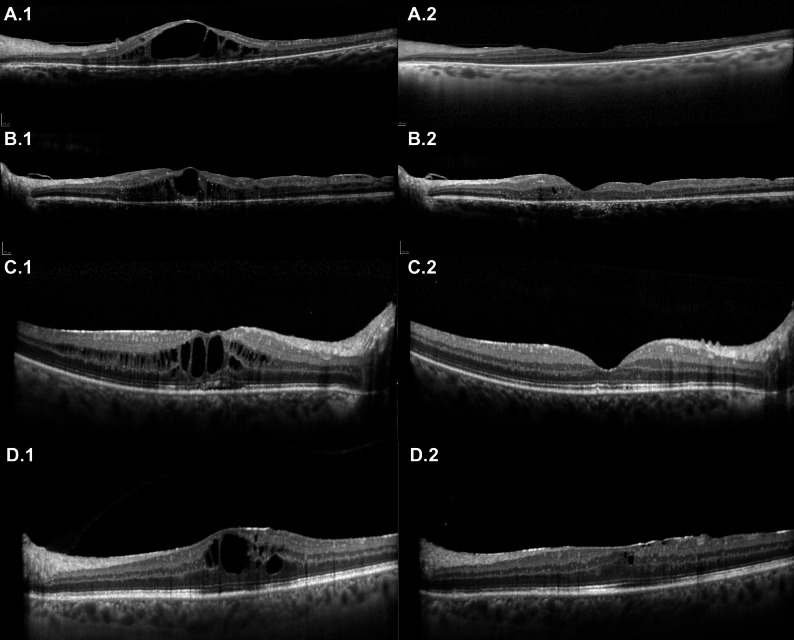
Macular OCT of 4 patients before and 24 months after FAi

A significant reduction in the number of recurrences per year was observed after FAi (4.62 ± 1.9 before vs. 0.31 ± 0.5 after, Wilcoxon Signed-Rank Test, *p* = 0.001). In all eyes, central retinal thickness (CRT) improved after FAi. Overall, central retinal thickness significantly decreased after FAi (474 ± 96 μm before vs. 305 ± 58 μm after, Wilcoxon Signed-Rank Test, *p* = 0.002) (Fig. 1). After FAi implantation, complete resolution of cystoid macular edema was achieved in 10 of 13 eyes (77%), while 3 eyes (23%) showed partial but clinically meaningful improvement. Among partial responders, CRT decreased from a mean of 515 ± 82 μm before FAi to 365 ± 47 μm at final follow-up (mean reduction: 150 ± 35 μm). All partial responders had previously shown a temporary but incomplete response to DEXAi or STTA, consistent with a chronic, relapsing pattern rather than true corticosteroid resistance. Figure 2 depicts examples of the macular OCT of 4 patients before and 24 months after FAi, demonstrating the anatomical response after the FAi.

Following FAi, macular edema relapsed in 4 eyes, at 28, 30, 30 and 36 months after the injection, respectively, and these were the only patients requiring local top-up therapy during follow-up. This represents a 36% relapse in the total of 11 patients that were under follow-up for more than 24 months. No relapses were observed before that time-point.

**Fig. 3:**
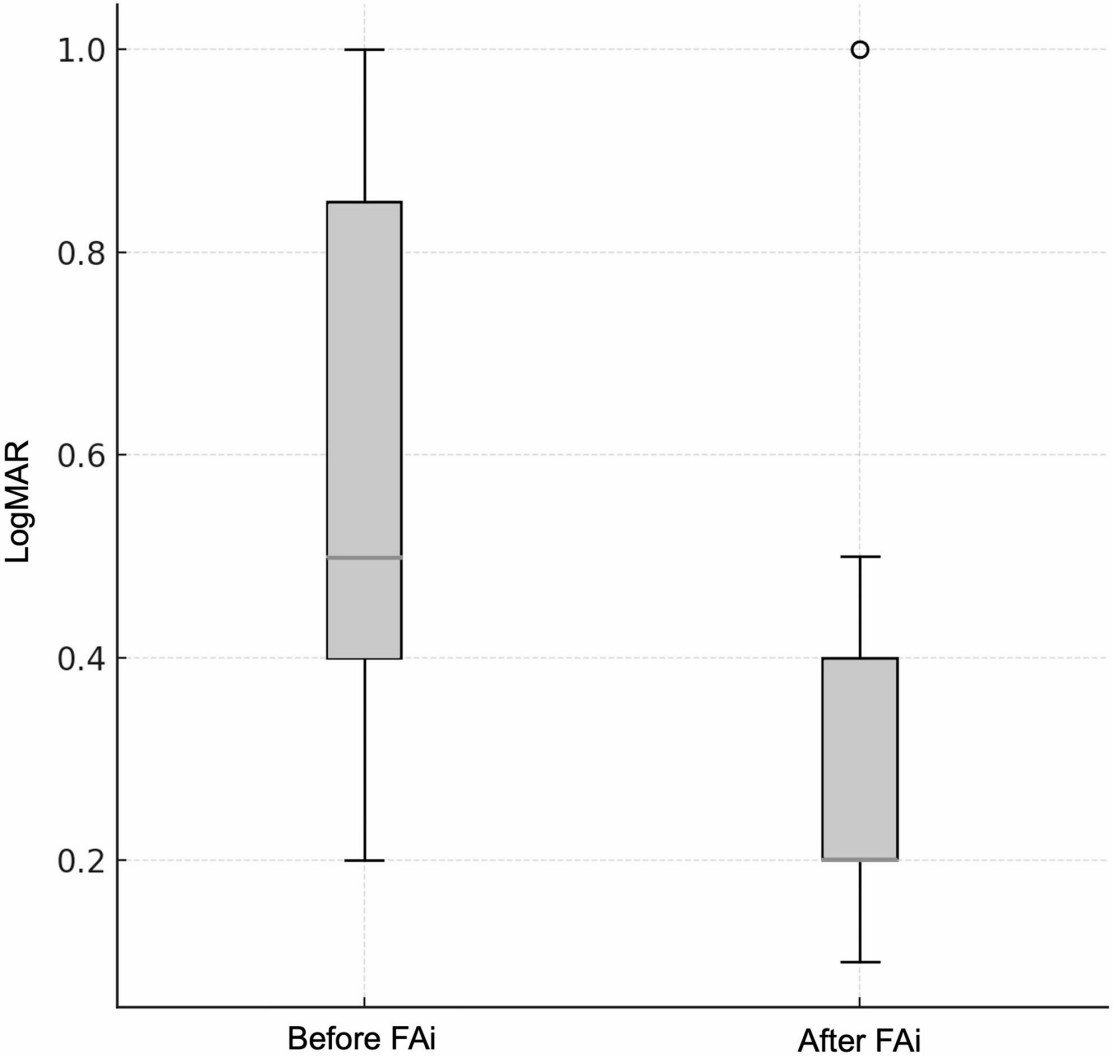
Boxplot of visual acuity before and after intravitreal 0.19 mg fluocinolone acetonide implant (FAi) (LogMAR)

Ten eyes (76.9%) showed BCVA improvement (1–4 lines), and none lost visual acuity (Fig. 3). The remaining 3 eyes maintained visual acuity before and after FAi.

IOP remained stable throughout all the follow-up period (before and after FAi). Five eyes were on topical IOP-lowering medication at the time of FAi; 2 required increased treatment, and 2 later underwent trabeculectomy. After FAi, only two eyes started IOP-lowering medication.

## Discussion

The purpose of this study was to evaluate long-term efficacy and safety of intravitreal 0.19 mg fluocinolone acetonide intravitreal implant (FAi) (Iluvien^®^) in non-infectious uveitic macular edema and compare it with previous sub-Tenon’s capsule triamcinolone acetonide (STTA) injection and/or intravitreal dexamethasone implant (DEXAi). In this case series with long-term follow-up, FAi demonstrated to be effective, safe, and to reduce recurrences compared to other local corticosteroids.

UME represents the most common cause for visual loss among uveitis patients and can occur secondary to any intraocular inflammatory condition [15, 16]. In our cohort, there is a very balanced distribution between all uveitic anatomic subgroups (anterior uveitis, intermediate uveitis, panuveitis and vasculitis). This is a very important information to account as it informs about the effectiveness of the FAi across many inflammatory subtypes. Almost all cases in our cohort presented idiopathic intraocular inflammation.

The management of UME may be challenging, due to its refractory nature. Both STTA, DEXAi and FAi are corticosteroid-based treatments used to control macular edema, but they differ significantly in terms of mechanism of action, duration of effect, efficacy, side effects, and administration technique [17–19].

STTA is a depot injection delivered in the sub-Tenon’s space with a rapid onset of action but relatively short duration of treatment, with efficacy declining over weeks/months, often requiring repeated injections every 2–3 months. Leder HA et al. determined that ME recurred in 53% of patients after the first periocular triamcinolone acetonide (TA) injection after a median of 20.2 weeks [17]. Intravitreal corticosteroid implants provide sustained release of steroids facilitating regression of ME with the advantage of less frequent injections. Real-life studies have demonstrated that dexamethasone implant ensures efficacy for a period of up to 4–6 months, with most cases requiring a reinjection after that period [20, 21].

The decline in efficacy of these treatment options, both STTA and DEXAi, in a few months resulted in fluctuations in inflammation control. All patients in our cohort were previously treated with STTA and more than 80% had been treated with DEXAi. Despite this treatment, patients presented a mean of 4 recurrences of ME per year, leading to subsequent STTA or DEXAi administration aiming to eliminate the retinal fluid.

After the switch to FAi, the number of recurrences per year dropped significantly (*p* < 0.001). Pavesio C et al. demonstrated that eyes with noninfectious posterior uveitis that received the FAi experienced a delayed onset of observed recurrence and a lower rate of recurrence of uveitis [22]. Pavesio and Heinz studied patients with bilateral non-infectious uveitis of the posterior segment, comparing FAi-treated (the more severely affected eye) and fellow eyes over 36 months, demonstrating that more FAi-treated than fellow eyes remained recurrence-free (28.8%) [6, 23].

Macular edema relapsed in 30.8% of patients, but the recurrences only occurred between months 28 and 36 after FAi injection. This result is aligned with the recognized duration of effect of the FAi, that maintains a sustained-release steroid delivery for approximately 30–36 months [24]. It represents an advantage comparing to other peri or intraocular steroids that maintain their effect typical over 3–6 months. In our cohort, the 4 cases that relapsed ≥28 months after FAi were the only patients requiring additional top-up or rescue local therapy, which represent another important finding supporting the effectiveness of the long-acting FAi. Preventing recurrent episodes of inflammation is very important in order to avoid cumulative damage related to each uveitis episode, that can ultimately lead to irreversible consequences [7, 25].

Our results corroborate the efficacy of the FAi in UME, both concerning anatomical and visual outcomes – with a decrease in central retinal thickness (CRT) and a visual gain. These efficacy results are in line with other studies in the literature. Studsgaard A et al. reviewed 20 patients with chronic non-infectious uveitis with associated cystoid macular edema over 2 years with BCVA improvement and CRT decrease [11]. Weber et al. evaluated 11 eyes with UME over 19 months documenting improved CRT and BCVA and a manageable safety profile [12]. Jabbour M et al. also established efficacity and safety of FAi during a follow-up of 12 months [1]. None of these case series compare the effectiveness of FAi with other steroid treatment (including STTA and DEXAi), which implies a very significant advantage of our study in the field of uveitic macular edema management.

Safety endpoints evaluated in our study after FAi intravitreal injection included intraocular pressure (IOP) elevation and cataract progression. FAi has a substantial risk of IOP elevation, with a gradual onset and often managed with IOP-lowering drops or surgery, if needed [26]. Nevertheless, both STTA and DEXAi can be associated with steroid induced IOP elevation, and a careful IOP monitoring is recommended in every patient under steroid treatment, even eyes with previously normal IOP [27]. As FAi represents a one-time treatment with no systemic side effects, is counteracts the disadvantage of repeated intravitreal injections that ultimately increase the risk of infections and ocular hypertension over time [6, 28].

Regarding the risk of progression of cataract, it must be considered in every patient under steroid treatment, regardless the type of administration. Several studies indicate cataract progression over 6–14 months, with posterior subcapsular cataract representing the most common diagnosis [29, 30]. In our cohort, all patients were pseudophakic previously to the FAi implant. The timing of cataract surgery related to the DEXAi or STTA administration was not evaluated in our study.

In patients with non-infectious uveitis requiring systemic immunosuppressive therapy for effectively control the intraocular inflammation, the FAi implant seems to be a very important therapeutic option for maintaining the disease under control while avoiding possible systemic side effects of the IMT. There is evidence suggesting that this treatment may enable the discontinuation of systemic anti-inflammatory therapy. In our cohort, after FAi 3 patients discontinued systemic immunosuppressive therapy (two patients with idiopathic panuveitis and one with idiopathic vasculitis), and none started new one. Studsgaard A et al. published a case series with 2 years of follow-up, concluding that FAi may offer reduction or cessation of local or systemic anti-inflammatory treatment (both systemic corticosteroids and other classes of immunomodulatory drugs) [11]. Moll‑Udina A et al., in a real‑world study of 22 patients with NIU and uveitic macular oedema treated, concluded that FAi implant can be considered as a single therapy in patients with NIU not associated with systemic disease, but should be considered as an adjuvant therapy for the management of ocular complications in cases with NIU associated with a systemic condition [8, 31]. Regarding the efficacy of FAi according to the type of uveitis, our cohort includes mostly cases of idiopathic inflammation, and it was not possible to identify any type of uveitis unresponsive to FAi. This is in line with another study in the literature that demonstrated improvements across all uveitis indications after FAi (including chronic panuveitis, retinal vasculitis/vitritis, pseudophakic CME/panuveitis and multifocal choroiditis) [9, 32].

Fluocinolone acetonide intravitreal implant represents a very important option, effective and safe, in the long-term treatment of chronic patients with uveitic macular edema. It proved to reduce the number of recurrences per year, decrease CRT and increase visual acuity – all outcomes achieved with a single intravitreal injection that demonstrated sustained efficacy over time, rather than with frequent treatments of shorter duration of action, with a similar safety profile. The need for multiple injections represents a significant burden for patients with associated risks and an important impact in the quality of life [10, 14, 33]. STTA remains a treatment modality of choice in patients with acute UME flare-ups (top up treatment), intolerance or contraindication to intraocular steroids (including aphakic eyes or eyes with scleral fixation intraocular lenses) or phakic patients concerned about cataract development. Burkholder BM et al. published the practice patterns and perceptions of uveitis specialists regarding the use of DEXAi in noninfectious uveitis. Among the inquired experts, UME was the most common indication for DEXAi, except in aphakic or advanced glaucoma patients [34]. A careful patient selection is always mandatory in chronic patients, especially considering the long duration inherent to the FAi, treatment burden of multiple injections and potential side effects of the different options available.

Regarding limitations, the small sample size of our cohort limits the statistical analysis power. The fact that this work only includes patients from a single tertiary center may eventually represent some bias due to local management protocols and the existence of access restrictions to different and new therapeutic modalities across different centers (especially considering more expensive therapies). Nevertheless, this study presents a very long follow-up length of patients with UME under steroid treatment, providing important insight regarding the real-world effectiveness of FAi implant in this very specific group of patients diagnosed with intraocular inflammation. This work also represents the first to our knowledge comparing the outcomes of FAi with other previously standard steroid therapy – sub-Tenon’s capsule triamcinolone acetonide injection and intravitreal dexamethasone implant.

## Conclusion

In this case series with long-term follow-up, 0.19 mg FAi for non-infectious UME was effective, safe, and reduced recurrences compared to other local corticosteroids. This treatment may enable the reduction or discontinuation of systemic anti-inflammatory therapy.

## Data Availability

The datasets used and/or analyzed during the current study are available from the corresponding author on reasonable request.

## Abbreviations

BCVA: Best-corrected visual acuity
CCT: Corticosteroids
CMT: Central macular thickness
CRT: Central retinal thickness
DME: Diabetic macular edema
DEXAi: Intravitreal dexamethasone implant
FAi: Intravitreal 0.19 mg fluocinolone acetonide implant
IOP: Intraocular pressure
IQR: Interquartile range
SD: Standard deviation
SD-OCT: Spectral domain optical coherence tomography
STTA: Sub-Tenon’s capsule triamcinolone acetonide
UME: Uveitic macular edema
